# Dorsolateral prefrontal transcranial magnetic stimulation in patients with major depression locally affects alpha power of REM sleep

**DOI:** 10.3389/fnhum.2013.00433

**Published:** 2013-08-02

**Authors:** Maria Concetta Pellicciari, Susanna Cordone, Cristina Marzano, Stefano Bignotti, Anna Gazzoli, Carlo Miniussi, Luigi De Gennaro

**Affiliations:** ^1^Cognitive Neuroscience Section, IRCCS Centro San Giovanni di Dio FatebenefratelliBrescia, Italy; ^2^Department of Psychology, University of Rome SapienzaRome, Italy; ^3^Department of Clinical and Experimental Sciences, National Institute of Neuroscience, University of BresciaBrescia, Italy

**Keywords:** major depression, repetitive transcranial stimulation, REM sleep, dorsolateral prefrontal cortex, alpha activity

## Abstract

Sleep alterations are among the most important disabling manifestation symptoms of Major Depression Disorder (MDD). A critical role of sleep importance is also underlined by the fact that its adjustment has been proposed as an objective marker of clinical remission in MDD. Repetitive transcranial magnetic stimulation (rTMS) represents a relatively novel therapeutic tool for the treatment of drug-resistant depression. Nevertheless, besides clinical evaluation of the mood improvement after rTMS, we have no clear understanding of what are the neurophysiological correlates of such treatment. One possible marker underlying the clinical outcome of rTMS in MDD could be cortical changes on wakefulness and sleep activity. The aim of this open-label study was to evaluate the efficacy of a sequential bilateral rTMS treatment over the dorsolateral prefrontal cortex (DLPFC) to improve the mood in MDD patients, and to determine if rTMS can induce changes on the sleep structure, and if those changes can be used as a surrogate marker of the clinical state of the patient. Ten drug-resistant depressed patients participated to ten daily sessions of sequential bilateral rTMS with a low-frequency TMS (1 Hz) over right-DLPFC and a subsequent high-frequency (10 Hz) TMS over left-DLPFC. The clinical and neurophysiological effects induced by rTMS were evaluated, respectively by means of the Hamilton Depression Rating Scale (HDRS), and by comparing the sleep pattern modulations and the spatial changes of EEG frequency bands during both NREM and REM sleep, before and after the real rTMS treatment. The sequential bilateral rTMS treatment over the DLPFC induced topographical-specific decrease of the alpha activity during REM sleep over left-DLPFC, which is significantly associated to the clinical outcome. In line with the notion of a left frontal hypoactivation in MDD patients, the observed local decrease of alpha activity after rTMS treatment during the REM sleep suggests that alpha frequency reduction could be considered as a marker of up-regulation of cortical activity induced by rTMS, as well as a surrogate neurophysiological correlate of the clinical outcome.

## Introduction

Major depression disorder (MDD) is considered not only a remarkable affliction for patients but also an economic burden for modern society (Luppa et al., 2007). The World Health Organization estimates that about 350 million people globally are affected by depression and that by 2030 it will rank as the largest contributor to disease. MDD represents a syndrome that encompasses many different symptoms which brutally affect several domains of the patient's life, with specific disturbances in mood, cognition and physical status. Among the physical symptoms, sleep alterations represent one of the most disabling manifestations of MDD and an early marker of the depression recurrence onset (Perlis et al., 1997; Baglioni et al., 2011). Insomnia, delayed sleep onset, non-restorative sleep with frequent awakenings during the night, and shorten duration of sleep are the most reported subjective complaints of MDD patients. Besides clinical observation, an altered macro- and micro-architecture of sleep characterizes the electroencephalographic (EEG) recordings of those patients (Lustberg and Reynolds, 2000; Armitage, 2007; Steiger and Kimura, 2010). An increase of rapid-eye-movement (REM) density, an increase of fast-frequency EEG activity, a prolongation of the first REM sleep period (Lauer et al., 1991; Riemann et al., 1994) with a reduction of sleep efficiency, an increase in REM sleep latency (Reynolds and Kupfer, 1987) and EEG slow-wave activity during non-REM sleep (NREM) (Mayers and Baldwin, 2005) represent the main neurophysiological correlates underlying subjective sleep complaints in the MDD.

Among EEG sleep disturbances, the REM sleep alterations are considered not only the main feature that characterize the sleep of depressed patients (Kupfer, 1976) as their adjustment are even used as biomarkers of disease remission. In that regard, several studies reported how antidepressant treatments have a clear-cut and immediate effects on REM sleep (Reynolds and Kupfer, 1987; Sandor and Shapiro, 1994; Sharpley and Cowen, 1995; Riemann et al., 2001), in terms of latency increase (Kupfer et al., 1981) and density decrease (Buysse et al., 1999), whereas the action on non-REM sleep are quite inconsistent (Sharpley and Cowen, 1995). Those data support the hypothesis of a specific link between REM sleep and depression.

Aiming at facing the drug-resistance in MDD, several non-pharmacological interventions have been proposed, including brain stimulation techniques [i.e., electroconvulsive therapy (ECT), deep brain stimulation (DBS)].

In the last decade, due to its ability to stimulate focally and non-invasively cortical brain areas (George et al., 2013), repetitive transcranial magnetic stimulation (rTMS) has been introduced for the treatment of medication refractory depression. TMS can induce a transient and painless electro-magnetic field through the skull, allowing the depolarization of the cortical neurons under the stimulated scalp location, and into connected areas (Li et al., 2010). Specifically, rTMS allows to modulate the cortical excitability, in a specific frequency-manner, with changes that persist beyond the duration of rTMS application. If applied at high frequency (≥5 Hz), TMS has been shown to induce a cortical excitability increase, while at low frequency (≤1 Hz) it induces a cortical excitability decrease.

Functional neuroimaging studies suggested that the prefrontal cortex, with its specific involvement in mood regulation, should be the cortical target of rTMS treatment in depression (Padberg and George, 2009). A typical abnormality observed in the cortical activity of major depressed patients is represented by prefrontal cortex asymmetry, with hypoactivity in the left dorsolateral prefrontal cortex (L-DLPFC) and relative hyperactivity of the right DLPFC (R-DLPCF) (Drevets, 2000; Walter et al., 2007; Grimm et al., 2008). That characteristical activation pattern has been shown both by examining resting cerebral glucose metabolism and blood flow with PET (Drevets et al., 1997; Videbech et al., 2002) and with EEG (Bruder et al., 1997; Debener et al., 2000; Allen et al., 2004). In agreement with those findings, several studies support the hypothesis that rTMS can increase with high frequencies and dampening with low frequencies the DLPFC excitability, rebalancing the altered functioning of the L-DLPFC and R-DLPFC, respectively involved as neurophysiological mediators of positive and negative mood (Silberman and Weingartner, 1986; Davidson, 1992). The clinical efficacy of rTMS for the treatment of the MDD (George et al., 2013), has been validated utilizing both unilateral (Lisanby et al., 2009; Pallanti et al., 2010) and bilateral approach, i.e., low frequency right-sided and high frequency left-sided over DLPFC (Fitzgerald et al., 2006). The latter is considered more effective for the treatment of major depression due the potential additive effect of both frequency stimulations, provided sequentially (Blumberger et al., 2012).

Although the clinical outcome of rTMS in depressed patients is relatively established, few studies have investigated the EEG effects of a rTMS treatment in depressed patients, in terms of cortical frequency changes, on wakefulness (Funk and George, 2008; Valiulis et al., 2012) and sleep activity (Saeki et al., 2012). In a recent study, Saeki et al. (2012), focusing their attention on slow wave activity (SWA) and sleep spindle activity, highlighted a local increase of SWA during NREM sleep and no change in REM sleep parameters after five sessions of high frequency rTMS to the left DLPFC.

Considering the role of other frequency bands, specifically the alpha activity as a cortical hypoactivity marker of depression syndrome, we hypothesized that the study of sleep changes induced by rTMS on all frequency bands, could allow to identify the biomarkers of the clinical remission and to better disclose the sleep neurophysiological correlates which could contribute to the improvement in clinical outcome observed in depressed drug resistant patients.

In the present open-label study, we aimed at verifying three outcomes. First, we wanted to evaluated the efficacy of a 10-days sequential bilateral rTMS treatment over the DLPFC to modulate mood in the MDD drug resistant patients, as assessed by the Hamilton Depression Rating Scale (HDRS). The second aspect was to evaluate if it was possible to induce changes on macro and micro-structure of sleep by means of rTMS, assessed by scoring EEG sleep recordings. Finally, we aimed at exploring the possible correlation between the clinical outcome and the neurophysiological changes.

According to these aims, sequential bilateral rTMS to DLPFC in treatment-resistant depressed patients, with a low-frequency TMS (1 Hz) over R-DLPFC and a subsequent high-frequency (10 Hz) TMS over L-DLPFC was applied. Spatial changes of EEG frequency bands were investigated by comparing maps not only of all EEG power bands but also of Hz-by-Hz EEG power values before and after rTMS treatment, during both NREM and REM sleep. The choice to investigate the cortical topography of REM and NREM sleep separately was dictated by the interest to differentiate the neuromodulator effects induced by rTMS on these sleep stages, considering their specific involvement and reciprocal relationship in the pathophysiological changes of depression (Armitage, 2007). Finally, the magnitude of the relative EEG sleep changes after rTMS was correlated with the changes of HDRS with the aim to identify if EEG sleep changes can be considered a biomarker of clinical outcome.

## Materials and methods

### Patients

Ten patients (5 males and 5 females, of mean age 52.8 ± 6.3 years, range 42–63 years) were included in this study. All of them had a diagnosis of MDD, formulated by an expert psychiatrist on the basis of a structured clinical interview for DSM-IV Axis I (SCID-I) according to the diagnostic and statistical manual of the American Psychiatric Association (APA. Diagnostic and statistical manual of mental disorders. 4^th^—TR ed. Washington, DC: APA Press; 2000). The MDD patients had no psychiatric comorbidity according to DSM-IV criteria, history of neurological disorders, epilepsy or substance abuse and contraindication for TMS (Rossi et al., 2009). For none of them the previous clinical outcome showed any improvement following the pharmacological antidepressant treatment. The pharmacological treatments are detailed in Table 1.

**Table 1:** **Concomitant pharmacological medications are detailed for all patients**.

**Pharmacological treatment**	**Patients (***n*** = **10**)**
BZD + SNRI	1
BZD + SNRI + Aneuro	3
BZD + SNRI + Tneuro	1
BZD + SNRI + TCA	1
BZD + SNRI + TCA + Aneuro + MS	1
BZD + TCA + MS	1
BZD + SSRI + SARI + Aneuro	1
SSRI + Tneuro	1

BZD, benzodiazepines; Antidepressants: SSRI, selective serotonin reuptake inhibitors; SNRI, serotonin-noradrenalin reuptake inhibitors; SARI, serotonin2-antagonists/serotonin reuptake inhibitors; TCA, tricyclic antidepressants; Antipsychotics: Tneuro, typical neuroleptic; Aneuro, atypical neuroleptic; MS, mood stabilizer.

Patients with primary sleep disorders (International Classification of Sleep Disorders, 2nd Edition- ICSD-2, 2005) as evaluated by polysomnographic recordings during an adaptation night in our laboratory, were excluded from the study.

Additional inclusion criteria were: a score ≥17 at the 21 items HDRS, or clinical improvement ≤50% obtained on the HDRS obtained and absence of improvement at the Clinical Global Impression (CGI) during the drug treatment with at least two classes of antidepressant drugs in standard doses for a period of at least 2 months. Prior to the inclusion, all patients had had a period of constant medication for 2 months and continued the same medication at the enrolment and for the full duration of the study. Therefore, pharmacological dosages were kept constant during the treatment. None of the patients received additional non pharmacological treatments, such as psychotherapy, during the study.

This study was approved by the CEIOC Ethics Committee of IRCCS Centro San Giovanni di Dio Fatebenefratelli, Brescia, Italy. Prior the beginning of the study and after a complete description of it, written informed consent was obtained from each patient.

### Procedure

None of the patients was familiar with the rTMS procedure. An open-label experimental design was used in the present study. Therefore, before the real treatment, each patient was subjected to a 3 days administration of a sham rTMS treatment applied over the parietal cortex, to allow their adaptation to the experimental setting (specifically, to the noise of rTMS). The real rTMS treatment consisted of 10 daily sessions of stimulation (Monday through Friday), for two weeks. Each subject underwent the stimulations at the same time each day, in the late afternoon. Before the real rTMS treatment and the following day the end of the whole treatment, the clinical symptoms were evaluated by means of the HDRS. An expert psychiatrist blind to the treatment performed the clinical rating. As reported above, the pharmacological treatment was kept constant during the rTMS treatment.

Each patient participated in the study for five nights. The sleep recordings, carried out in a sound-proof room, were scheduled in the adaptation night followed by a baseline night (BSL) in correspondence respectively of the second and third session of sham pre-treatment, and three nights during the real rTMS treatment, respectively after the first (R1), the ninth (re-adaptation night—R2) and tenth (final treatment—R3) rTMS session. Before the sleep recordings, all patients were stimulated 60 min before light off. Time in bed after lights off was ~7 h.

### Transcranial magnetic stimulation

rTMS was delivered by a Magstim Super Rapid Magnetic Stimulator (50 Hz—biphasic, four boosters) with a standard double 70-mm coil (Magstim Company Limited, Whitland, UK). We alternated two coils, in order to allow cooling during the repetitive stimulation without interruption. Before starting the rTMS treatment, the motor threshold (MT) was determined for each subject, respectively over the left and right motor area, following international standards (Rossini et al., 1994).

Aiming to align all patients into a balanced baseline state, a sham rTMS treatment was employed in the 3 days before real rTMS. For sham treatment, a 25-mm thick plywood shield, build to appear as an integral part of the apparatus, was interposed between the coil itself and the scalp, separating the two. Moreover, the ventral surface of the coil, from which the magnetic field was delivered, was upside down and the stimulus intensity was at 110% of MT. In the sham session, 1200 pulses at 1 Hz frequency for 20 s, with an inter-train interval of 10 s, were delivered over the parietal cortex. The real rTMS treatment was delivered at 110% of MT on the frontal scalp area overlying the R-DLPFC and L-DLPFC. For the identification of the stimulated brain areas, the left and right DLPFCs were localized on the basis of a reconstruction of cerebral cortex in the Talairach coordinate system using the SofTaxic neuronavigation system (EMS, Bologna, Italy www.softaxic.com). Using this system, we marked the stimulation sites over the left and the right DLPFC, coinciding with Brodmann areas 46. These localizations were in accordance to previous reports (e.g., Saeki et al., 2012).

The coil was placed tangentially to the scalp with the handle pointing backwards and laterally at about a 45° angle away from the midsagittal axis of the patient' heads and oriented to elicit a postero-lateral-anteromedial current flow in the brain tissue. The rTMS was first applied at low frequency over the R-DLPFC and then at high frequency over the L-DLPFC.

Low frequency rTMS consisted of 60 trains of stimuli at 1 Hz frequency for 20 s with an inter-train interval of 10 s. High frequency rTMS consisted of 60 trains of stimuli at 10 Hz frequency for 2 s, with an inter-train interval of 28 s. An interval of ~30 min separated the two sessions. Each session lasted ~30 min and the total number of pulses was the same between the two stimulation paradigms (in total 1200 pulses both for the 1 Hz-TMS and that for the 10 Hz-TMS). These parameters are in line with safety recommendations for rTMS (Wassermann, 1998; Wassermann and Lisanby, 2001).

### Polysomnographic recordings

A BrainAmp Recorder System (BrainAmp 32 channel, BrainProducts GmbH, Munich, Germany) was used for polygraphic recordings. EEG signals were high pass filtered with a time constant of 0.3 s and low pass filtered at 70 Hz, and digitized at a sampling rate of 250 Hz. The nineteen unipolar EEG derivations of the international 10–20 system (Fp1, Fp2, F7, F8, F3, F4, Fz, C4, C3, Cz, P3, P4, Pz, T4, T6, T3, T5, O1, O2) were recorded from scalp electrodes. Additional electrodes were used as the ground and reference. The ground electrode was placed in the midfrontal (Fpz) position. The reference electrode was placed from a left mastoid, while recordings obtained from right mastoid electrode was used off-line to re-reference the scalp recordings. Ten additional unipolar EEG derivations were recorded. One electrode was positioned exactly over the individual right DLPFC (R-DLPFC), and other four electrodes were placed around this point in each orthogonal direction at a distance of 1 cm. The R-hotspot (R-DLFPC) resulted very close to the F3 electrode of the 10–20 system. As a denotation, the four orthogonal positions will be indicated as “anterior to right dorsolateral prefrontal cortex” (aR-DLPFC), “medial to right DLPFC” (mR-DLPFC), “posterior to right DLPFC” (pR-DLPFC), and “lateral to right DLPFC” (lR-DLPFC). The same montage and denotation was also used for the left dorsolateral prefrontal cortex (L-DLPFC, aL-DLPFC, mL-DLPFC, pL-DLPFC, and lL-DLPFC). Horizontal and vertical eye movements were detected by recording the electro-oculogram (EOG) in order to monitor subject behavior on-line and reject, off-line, trials with ocular artifacts. The submental electromyogram was recorded with a time constant of 0.03 s. Bipolar horizontal eye movements were recorded with a time constant of 1 s. The bipolar horizontal electrooculogram was recorded from electrodes placed about 1 cm from the medial and lateral canthi of the dominant eye. Impedance for all electrodes was kept below 5 kΩ.

### Data analysis

#### Clinical data analysis

The effect of the rTMS treatment on the clinical outcome (HDRS) was assessed by means of an analysis of variance (ANOVA), in which the clinical data at BSL and at R3 nights were compared.

#### Sleep measures

Sleep stages of BSL and R3 nights were visually scored in 20 s epochs, according to the standard criteria (Rechtschaffen and Kales, 1968). The following were considered as dependent variables: (a) stage 1 latency; (b) stage 2 latency; (c) REM latency; (d) percentage of stage 1; (e) percentage of stage 2; (f) percentage of REM sleep; (g) wakefulness after sleep onset (WASO), expressed as the intra-sleep time (min) spent awake; (h) number of awakenings (the number of >10 s episodes of WASO); (i) number of arousals (the number of <10 s episodes of WASO); (j) total sleep time (TST), defined as the sum of time spent in stage 1, stage 2, SWS, and REM; (k) total bedtime (TBT); (l) sleep efficiency index (SEI = TST/TBT × 100). The polysomnographic EEG measures were submitted to one-way repeated measure ANOVAs, comparing BSL, and R3 nights.

#### Quantitative analysis of sleep EEG

The polygraphic signals (29 EEG channels, EOG, and electromyography) were low pass filtered at 30 Hz, analog to digital converted on-line with a sampling rate of 128 Hz, and stored on the disk of a personal computer. Ocular and muscle artifacts were excluded off-line by visual inspection. We investigated the 0.50–25.00 Hz frequency range, computing the power spectra by a Fast Fourier Transform routine for 4 s periodgrams. Before conducting the statistical analyses, the data were reduced to the traditional EEG bands of sleep, by collapsing adjacent 0.25-Hz bins: delta (0.50–4.75 Hz), theta (5.00–7.75 Hz), alpha (8.00–11.75 Hz), sigma (12.00–15.75 Hz), and beta (15.00–24.75 Hz). The power spectra were calculated separately for the NREM sleep (stage 2 + 3 + 4) and the REM sleep. These EEG power values were considered as dependent measures. The values were log-transformed, color coded, plotted at the corresponding position on the planar projection of the scalp surface, and interpolated (biharmonic spline) between electrodes.

The EEG power maps were computed separately for the BSL and the R3 nights, and separately for NREM and REM sleep. Then, power values of the BSL vs. R3 nights were compared for each band and scalp location by means of paired *t*-tests. Finally, the statistical maps of the *t*-value comparisons were color coded and plotted separately for NREM and REM sleep.

The Bonferroni correction for multiple comparisons was applied. Considering the mean correlation between the variables of the NREM sleep (*r* = 0.49), the alpha level was then adjusted to ≤0.0039 (*t* ≥ 3.84). Similarly, considering the mean correlation between the variables of the REM sleep (*r* = 0.57), the alpha level was then adjusted to ≤ 0.0059 (*t* ≥ 3.58).

## Results

### Clinical data

**Figure 1:**
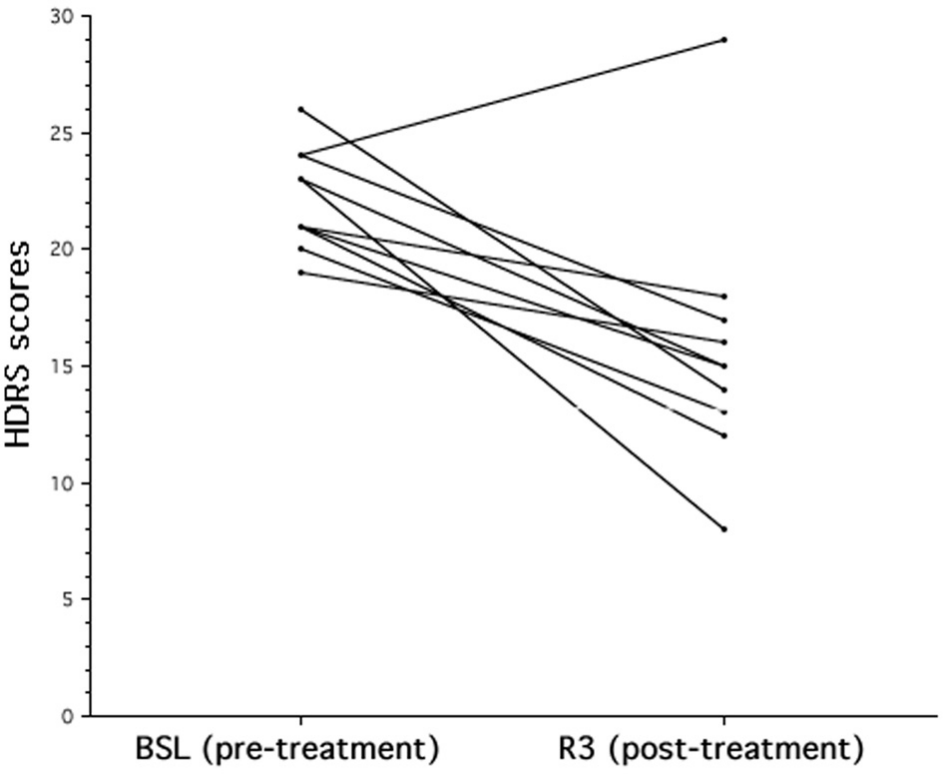
**The effects of rTMS on mood in patients with major depression**. Individual Hamilton Depression Rating Scale (HDRS) score evaluated at baseline (BSL) and after rTMS treatment (R3). After rTMS there is a significant decrease of HDRS score that corresponds to a clinical status improvement.

The analysis on the HDRS scores revealed a significant improvement of the clinical status as a consequence of the rTMS treatment [BSL = 22.2, ± 2.15; R3 = 15.7, ± 5.46; *F*_(1, 9)_ = 14.16; *p* = 0.0045]. As shown in Figure 1, nine out of ten patients improved at HDRS after the rTMS treatment, and their mean percentage decrease was 35.0% (±13%).

### Polysomnography

Table 2 reports the results of the analyses of variance on polysomnographic (PSG) variables. The macrostructural variables of sleep pointed to a lack of significant differences between baseline and post-treatment nights (R3), with the exception of a slight reduction of the time spent in WASO (BSL = 76.9 min, ± 54.4; R3 = 52.6 min, ±31.4). Notably, there was no change in the measures of latency and of time spent in the REM sleep.

**Table 2:** **Means and standard deviations (*SD*) of the polysomnographic variables, before (BSL) and after rTMS treatment (R3), based on visual scoring for all patients**.

**Variables**	**BSL**		**R3**		*****F***_**(1, 9)**_**	***P***
	**Mean**	***SD***	**Mean**	***SD***		
Stage 1 latency (min)	32.17	27.27	27.36	29.12	0.63	0.45
Stage 2 latency (min)	35.63	30.23	29.80	31.20	0.61	0.45
REM latency (min)	129.90	21.12	122.50	46.68	0.38	0.55
Stage 1 (%)	17.41	16.74	16.99	14.01	0.02	0.88
Stage 2 (%)	66.76	20.55	64.17	18.69	0.94	0.36
REM (%)	15.83	9.41	18.84	6.37	1.76	0.21
WASO (min)	76.88	54.44	52.62	31.35	4.09	0.07
Awakenings (#)	24.20	13.98	21.30	8.65	0.91	0.36
Arousals (#)	82.80	51.42	85.70	53.16	0.22	0.65
TST (min)	414.40	65.62	420.83	69.55	0.13	0.73
TBT (min)	573.23	148.67	501.97	36.98	2.47	0.15
SEI % (TST/TBT)	75.68	18.55	83.74	12.24	2.08	0.18

The results of one-way repeated measure analyses of variance are also reported. No significant difference is present between BSL and R3, with the exception of a slight reduction of wakefulness after sleep onset. REM, rapid eye movement; WASO, wake after sleep onset; TST, total sleep time; TBT, total bed time; SEI, sleep efficiency index.

### Quantitative analysis of EEG

#### NREM sleep

**Figure 2:**
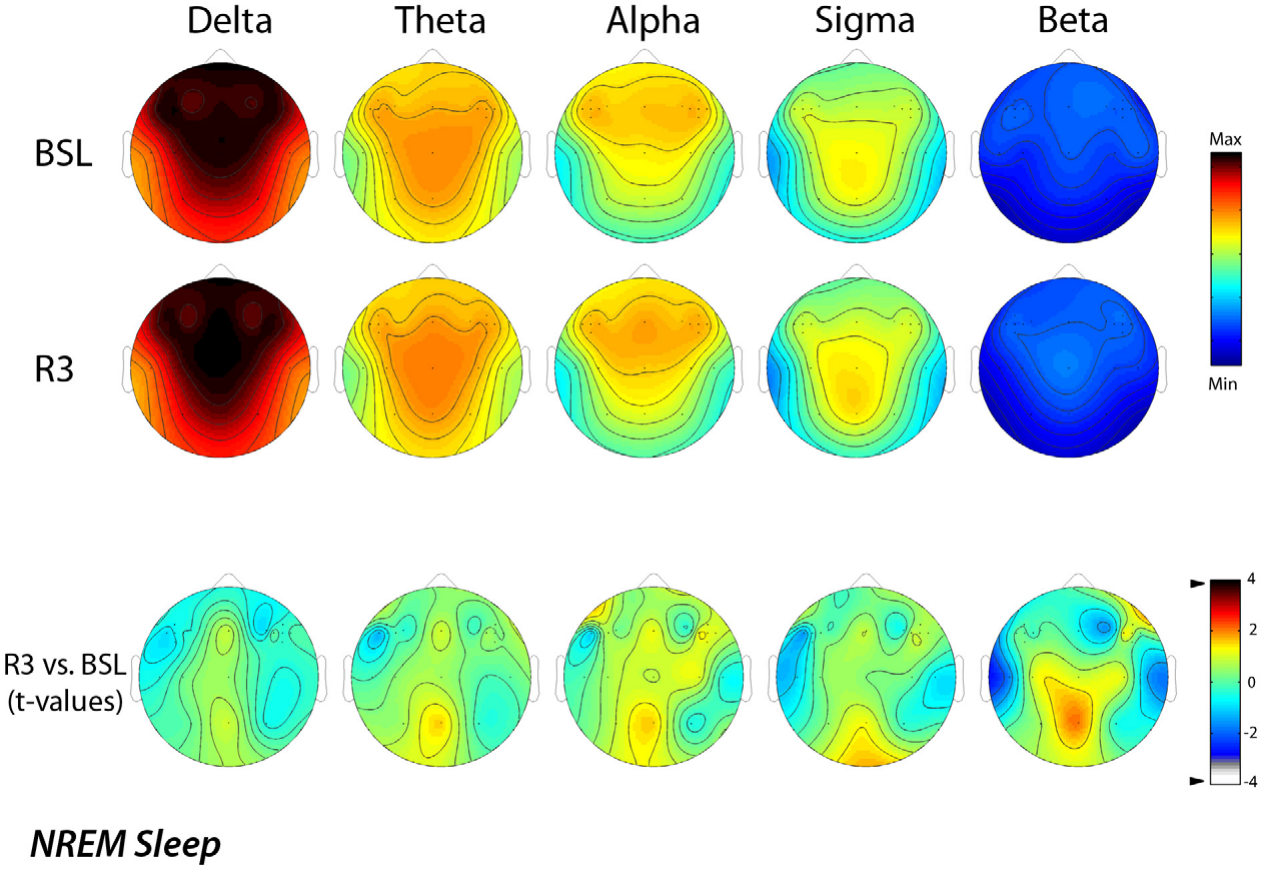
**Topographic distribution of EEG power during NREM sleep of the baseline night (BSL) and of the night after the rTMS treatment (R3)**. Average values are normalized by total power, color-coded, plotted at the corresponding position on the planar projection of the scalp surface and interpolated (biharmonic spline) between electrodes. The maps are based on the 19 unipolar EEG derivations of the international 10–20 system with averaged mastoid reference, and on 10 additional derivations, positioned in both hemispheres respectively over left and right dorsolateral prefrontal cortex and in each orthogonal direction at a distance of 1 cm from the hotspot (electrode positions indicated by dots). In the upper part, the first two rows show the absolute EEG power during NREM sleep of the baseline night (BSL) of the night after the rTMS treatment (R3). Maps are plotted for the following EEG bands: delta (0.50–4.75 Hz), theta (5.00–7.75 Hz), alpha (8.00–11.75 Hz), sigma (12.00–15.75 Hz), and beta (15.00–24.75 Hz). To optimize the contrast, each map was scaled separately between minimal (min) and maximal (max) power values. In the lower part, the statistical maps (*t*-values maps) of the comparison between R3 vs. BSL are illustrated for each frequency band (positive *t*-values indicate a prevalence of the first over the second term). Please note that the color code reports the actual *t*-values (the two-tailed level of significance of *p* = 0.0039, after the Bonferroni correction, corresponds to a *t* = 3.84).

Figure 2 shows the EEG activity in the NREM sleep, averaged over all the 29 scalp locations during baseline and post-treatment sleep. Power maps of both nights indicated stable patterns within different frequency ranges, and the data of maxima and minima exhibited the typical features of power spectra during the NREM sleep. The delta and alpha bands showed a frontal midline predominance and minimum values over the temporal regions. In the theta band, the highest values were at the fronto-central midline areas, while the sigma band showed a centro-parietal maxima. These topographical maps were substantially stable in the different conditions. The lower part of the figure reports the results of the statistical comparisons, and no significant difference was highlighted for any frequency band and scalp location as a consequence of rTMS sessions.

#### REM sleep

**Figure 3:**
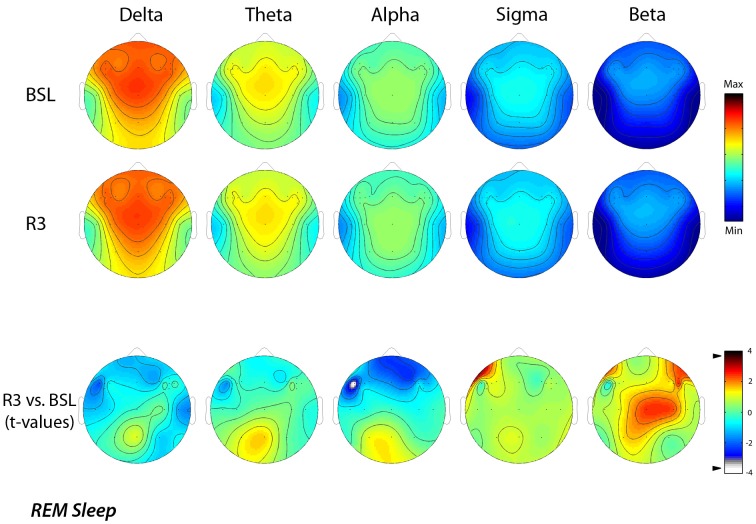
**Topographic distribution of EEG power during REM sleep of the baseline night (BSL) and of the night after the rTMS treatment (R3)**. Average values are normalized by total power, color-coded, plotted at the corresponding position on the planar projection of the scalp surface and interpolated (biharmonic spline) between electrodes. The maps are based on the 19 unipolar EEG derivations of the international 10–20 system with averaged mastoid reference, and on 10 additional derivations, positioned in both hemispheres respectively over left and t right dorsolateral prefrontal cortex and in each orthogonal direction at a distance of 1 cm from the hotspot (electrode positions indicated by dots). In the upper part, the first two rows show the absolute EEG power during REM sleep of the baseline night (BSL) and of the night after the rTMS treatment (R3). Each columns reports maps of the EEG power in the delta, theta, alpha, sigma, and beta frequency ranges. To optimize the contrast, each map was scaled separately between minimal (min) and maximal (max) power values. In the lower part, the statistical maps (*t*-values maps) of the comparison between R3 vs. BSL are illustrated for each frequency band (positive *t*-values indicate a prevalence of the first over the second term). Please note that the color code reports the actual *t*-values (the two-tailed level of significance of *p* = 0.0059, after the Bonferroni correction, corresponds to a *t* = 3.58). Specifically, the alpha power decreased significantly (*t* = −3.79; *p* = 0.0043) during REM sleep at R3 respect to BSL night on lL-DLPFC.

Similarly, Figure 3 shows EEG activity in REM sleep during the BSL and R3 nights. The same stable patterns within different frequency bands were roughly maintained in REM sleep, with the notable exception of the 8–15 Hz range. Topographical maps confirmed a prevalence of the delta band at frontal midline similar to NREM sleep, with minimum values over the temporal regions. In the theta band, the highest values were at the fronto-central midline areas, while the alpha and sigma bands showed a centro-parietal maxima. Both the sigma and beta activity showed minimal values in correspondence of the temporal sites.

The lower part of the figure reports the results of the statistical comparisons, which pointed to a significant decrease of the alpha power (*t* = −3.79; *p* = 0.0043) over the lL-DLPFC site in the R3 compared to the BSL night.

**Figure 4:**
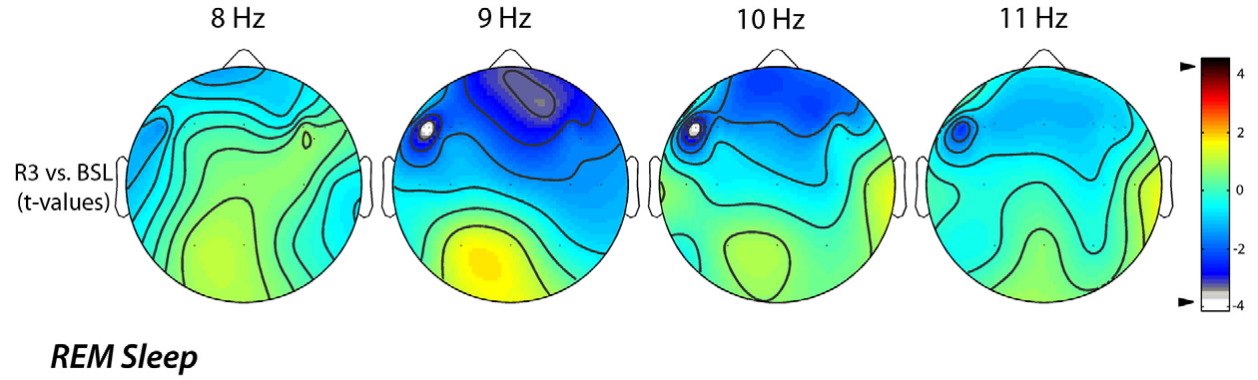
**Topographic statistical distribution of the change in alpha power (8.00–11.75 Hz), assessed by comparing (*t*-tests) R3 and baseline nights, for the REM sleep**. Values are expressed in terms of *t*-values: negative t-values indicate a decrease at R3 over the baseline night. Filled white circles indicate electrodes showing a significant difference. The analysis of EEG changes with a 1-Hz frequency resolution highlighted an alpha power decrease on lL-DLPFC both to 9 Hz (*t* = −4.14; *p* = 0.0025) and to 10 Hz (*t* = −4.12; *p* = 0.0026).

A further analysis of EEG changes with a 1-Hz frequency resolution, aimed to refine the significant difference found for the alpha band, clearly showed that the decrease of EEG activity during REM sleep, mainly regarded the 9 (9.00–9.75 Hz) and 10 (10.00–10.75 Hz) Hz bins. The statistical maps comparing the BSL and the R3 nights showed that even for those frequency bins the observed differences were significant (Figure 4). Specifically, we observed a significant decrease in the alpha band on lL-DLPFC, respectively at 9 Hz (*t* = −4.14; *p* = 0.0025) and at 10 Hz (*t* = −4.12; *p* = 0.0026).

### Relation between EEG changes and clinical improvement

**Figure 5:**
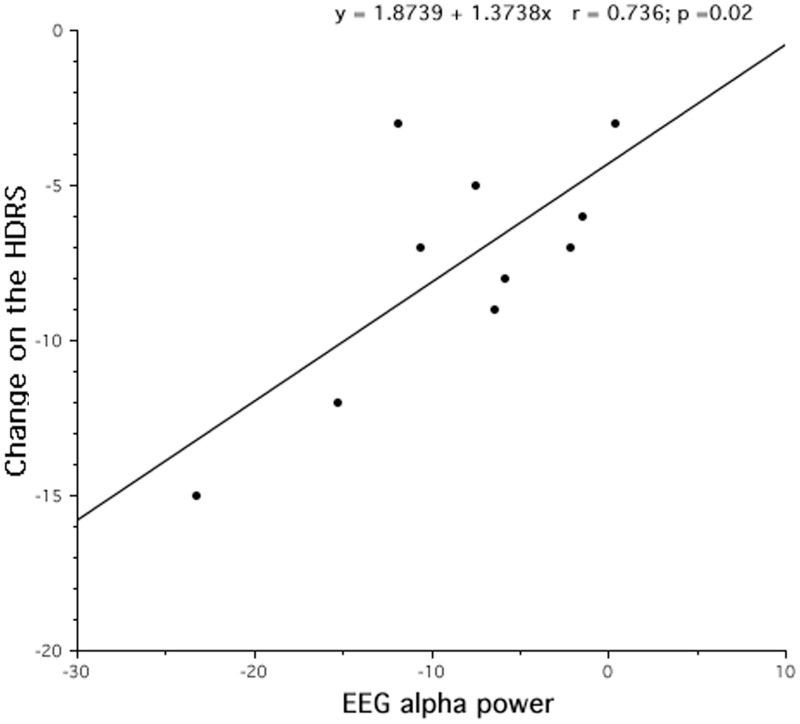
**Changes after rTMS treatment in REM alpha power and clinical improvement, assessed by Hamilton Depression Rating Scale (HDRS)**. After 10 days of treatment, the decrease of REM alpha power results strongly correlated to the treatment outcome.

According to the findings of the topographical analyses, the main questions remains: “Is the EEG difference associated to the extent clinical improvement?” For this reason, we assessed the correlation between the magnitude of decreased alpha activity in REM sleep at the lL-DLPFC site and the corresponding clinical improvement (in 9 out 10 patients), as expressed by the post-treatment minus pre-treatment HDRS scores. As shown in Figure 5, a significant correlation (*r* = 0.74), although on a small sample size, was highlighted with larger decreases of alpha activity in REM sleep associated to larger clinical improvements.

## Discussion

The present study was aimed at evaluating the clinical efficacy of a sequential bilateral rTMS treatment in patients with treatment-resistant MDD and to assess the rTMS-induced macro- and microstructural sleep changes. Keeping in mind the limitations of our open-label design, we observed that rTMS treatment with high frequency over L-DLPFC and low frequency over R-DLPFC was able to induce antidepressant effects. The application of rTMS induced a significant decrease of HDRS score, a clinical marker of mood improvement. Despite no changes detected in the sleep macrostructure after the treatment, a frequency-dependent rTMS effect was observed on the microstructural REM sleep pattern. Specifically, the rTMS induced an alpha power decrease in the REM sleep over the L-DLPFC, that resulted strongly correlated to the clinical outcome.

The observed clinical improvement was in accordance with previous studies that demonstrated the antidepressant efficacy of rTMS (George et al., 2000; Burt et al., 2002; Grunhaus et al., 2003; Fitzgerald et al., 2009). The inclusion of patients with a diagnosis of medication-resistant depression, in our study, could have limited the magnitude of the therapeutic response, as those patients show a lower response rate to antidepressant intervention compared to non-treatment-resistant patients (Fitzgerald, 2003; Fregni et al., 2006; Brakemeier et al., 2007; Padberg and George, 2009). Even if the mean HDRS response rate decrease was statistically significant while not clinically relevant, declining from very severe and severe depression before the treatment to moderate and mild depression after the treatment, our results should be interpreted positively as the mean percentage of improvements in HDRS score is 35% after rTMS in nine out of ten non-responders patients. To that regard, we hypothesized that the lack of clinical improvement of the non-responder patient to rTMS treatment could be due to his older age, considering the inverse relationship between age and antidepressant response (Manes et al., 2001; Fregni et al., 2006); however the direct assessment of the relation between older age and cortical atrophy has not been viable in the current protocol. The impact of atrophy, in fact, increasing the distance of the coil from the prefrontal cortex (Kozel et al., 2000) would decrease the strength of the induced electrical current and limit the effectiveness of treatment. Probably, a rTMS dose adjusted to overcome cortical atrophy or a more long TMS course (Lisanby et al., 2009) could have been more effective for that patient.

Our clinical results support the notion that the sequential bilateral rTMS combining high frequency left-side and low frequency right-side is an effective antidepressant treatment (Fitzgerald et al., 2006), even though by a point of view of sleep pattern we did not found any macrostructural correlate to that outcome. Considering the role of the REM sleep as a diagnostic and prognostic biological marker of depression, the absence of changes observed on the sleep macrostructure should be considered unexpected. Some changes on the REM variables after the rTMS treatment was expected in the direction of inhibition of REM sleep, manifested by an increase of its latency and a decrease of its amount. However, those changes are frequently observed in the between-subjects protocols that compare the data of depressed patients with those of healthy control subjects (Giles et al., 1993). We hypothesized that with the within-subjects protocol used in this study, it was unlikely to observe evident changes in sleep parameters, like the REM sleep latency or density, that could be well stabilized by depressive disorder, representing a “trait” characteristic (Rush et al., 1986; Giles et al., 1989; Tsuno et al., 2005; Rao and Poland, 2008; Pillai et al., 2011). Moreover, our results confirm the previous findings pointing out how some EEG sleep structural measures remain stable even after pharmacological (Steiger et al., 1989; Kupfer et al., 1994; Murck et al., 2003), non-pharmacological antidepressant treatment (Thase et al., 1998) or combined therapy (Giles et al., 1993).

Although we did not find any changes in the REM macrostructural variables, the main result of the present study regards a specific modulation of the cortical activity during the REM sleep. The observed decrease of cortical activity in the alpha band during the REM sleep after rTMS treatment could represent a specific microstructural marker of depression's remission.

From lesion and neuroimaging studies (Baxter et al., 1989; Martinot et al., 1990; Henriques and Davidson, 1991; Bench et al., 1992; Biver et al., 1994), it is well established as major depression is considered as a hypoactivity syndrome associated with reduced left prefrontal metabolism and cortical dysfunction, in terms of cortical activation decrease (Drevets, 2000; for a review see, Koenigs and Grafman, 2009). Several studies have reported that a marker of this dysregulation could be identified in the alpha power increase, given the inverse relationship between EEG alpha and neural activity, and the consequent role of alpha activity as an inhibitory oscillatory rhythm (Henriques and Davidson, 1990, 1991; Pollock and Schneider, 1990; Ricardo-Garcell et al., 2009; Kemp et al., 2010). Even though the lack of a control healthy group does not allow us to verify if our patients were characterized by a left hypoactivity syndrome (Lubar et al., 2003; Jaworska et al., 2012), our results can be explained by that model. Regarding the alpha power decrease observed during the REM sleep and localized over the left cortex stimulated with high frequency rTMS, we hypothesized that such rTMS frequency, increasing the cortical excitability, might act toward normalizing the DLPFC dysregulation, expressed by the cortical activity modulation observed during the REM sleep. That result is strongly in line with the increase of the cortical excitability after the high frequency rTMS (Fitzgerald et al., 2002; Hallett and Rothwell, 2011). Our results confirm the previous findings that the decrease of the frontal hypoactivity, probably in terms of cortical activity's normalization, could represent the underlying mechanism by which not only antidepressant drugs (Baxter et al., 1989; Mayberg et al., 2000) but also high frequency TMS fosters improvements in depressive disorder, as highlighted by the higher cerebral blood flow values and the cortical excitability increase (George et al., 1999, 2000; Teneback et al., 1999; Speer et al., 2000; Nahas et al., 2001; Rossini et al., 2010). We hypothesize that the high frequency rTMS, stimulating the underlying cortical region and changing the activity of the corresponding neural tissue, could determine an up-regulation of the local activity (Chen, 2000; Fitzgerald et al., 2002). Specifically, the antidepressant effect of the high frequency TMS could be the result of a re-activation of hypofunctional L-DLPFC, in terms of increase of cortical activity, and the neurophysiological biomarker of this effect could be objectified in the decrease of local alpha activity during REM sleep. Our result is strongly in line with the recent hypothesis that rTMS alters and resets cortical oscillators, regulating the intrinsic cerebral rhythms and restoring normal brain function (Leuchter et al., 2013). In particular, the high frequency rTMS could act as an exogenous input, affecting the cortical components, normalizing the intrinsic brain activity and ameliorating the depressive symptoms.

Moreover, the high frequency rTMS could modulate not only the local neuronal firing rate, depolarizing the excitability of neurons, but could also produce a therapeutic effect through the modulation of the monoaminergic receptors involved in the REM sleep. In that regard, an attractive hypothesis to explain both the antidepressant effects induced by rTMS and the REM sleep modulations could be identified in the involvement of serotoninergic mechanisms (Cohrs et al., 1998; Ben-Shachar et al., 1999; Gur et al., 2000; Post and Keck, 2001; Adrien, 2002; Padberg and George, 2009; Baeken et al., 2011). These mechanisms are the same that are involved in other pharmacological (Maudhuit et al., 1997) and non-pharmacological (i.e., total or REM sleep deprivation) (Prevot et al., 1996) antidepressant interventions.

Regarding the bidirectional relationship between sleep disturbance and depression (Riemann et al., 2001; Tsuno et al., 2005) and even though the connection between sleep modifications and clinical improvement have not yet been established, two possible scenarios could be hypothesized to explain our results. In the first one, the rTMS treatment could have acted modulating the clinical symptoms and their improvement could causally affect the EEG sleep topography. In the second one, the rTMS-induced effects on sleep cortical activity could represent the physiological framework, underlying the clinical outcome. Although our results do not allow us to clearly discriminate whether the alpha activity change observed during REM sleep after rTMS treatment was the trigger for the clinical improvement or only an its passive consequence, we speculate that the topographical sleep change was causally determined by the clinical improvement induced by rTMS treatment, representing a neurophysiological correlate of clinical outcome.

The main neurophysiological results of the present study, namely an alpha decrease during the REM sleep in the same frequency range of high frequency rTMS applied in this protocol (i.e., 10 Hz) allow us to speculate that high frequency rTMS could act by saturating the local alpha power and producing an excitability increase functional to remission of the depressive disorder. A similar change in the alpha band was observed in REM sleep after a protocol of sleep deprivation (Roth et al., 1999; Marzano et al., 2010), supporting such frequency as a quantitative and distinctive marker of REM sleep. In that regard, we speculate that the alpha frequency decrease could represent a biological marker of the sleep pressure for the REM sleep, as the delta power represents a well-recognized index of sleep homeostasis for the NREM sleep (Tononi and Cirelli, 2006).

Another important issue is the topography-specificity of the alpha change between the stimulated DLPFCs. Even though no sleep frequency changes were observed over the right stimulated cortex, we speculate that the topographical effect over the L-DLPFC didn't exclude a sequential effect of low frequency rTMS applied over the R-DLPFC cortex, most probably through trans-synaptic connections (Paus et al., 1997; Nahas et al., 2001). Probably, the application of low frequency before the high frequency rTMS may have contributed to prime the effects observed over the left cortex, determining a synergetic effect on the clinical outcome (Fitzgerald et al., 2006) even though no detectable at cortical level.

Finally, even though our study was focused on all EEG frequency bands, our results are partially in agreement with the only study in which the sleep electrophysiological effects induced by rTMS treatment on patients with a major depressive episode were investigated (Saeki et al., 2012). Regarding NREM sleep, in this latter study a delta power increase at left DLFPC was observed after the initial five rTMS sessions, but not after the total 10 rTMS sessions. In agreement to these authors, also our negative finding on SWA during NREM sleep after 10 rTMS sessions could be due to the involvement of homeostatic regulation mechanism. On the other hand, the discrepancy of results during REM sleep, when we observed a local decrease of alpha activity, could be due to their focus on two specific EEG frequency bands (slow wave and spindle activity), not considering all sleep bands.

Several limitations need to be addressed. The first limitation was the experimental design. The open-label experimental design and the lack of a sham comparison group (with subsequent sleep recordings) doesn't allow us to thoroughly exclude a placebo effect in the clinical response of our patients (Miniussi et al., 2005), though the observed linear relationship between EEG and HDRS changes suggests a specificity of the relationship.

Moreover, our sample size was relatively small and the patients were all under pharmacological therapy during rTMS treatment, making it impossible to evaluate the clinical and neurophysiological effect of rTMS as stand-alone treatment. In that regard, the non-suspension of the drug treatment could be responsible for the lack of significant macrostructural differences, highlighted both in the REM (in terms of latency and density) and the NREM sleep. Specifically, it is well known how the REM sleep duration is reduced by the antidepressant drug action until to its suppression (Holshoe, 2009), and how the NREM sleep, and in particular the sigma band and SWA are affected by benzodiazepines (e.g., Bastien et al., 2003). Only additional studies with a wider patient sample and patients pharmacological washing out, as well as a sham condition may allow a better understanding of the macro- and microstructural effects induced by rTMS treatment on depressive disorder.

## Conclusion

Concluding, in line with the theory of the left frontal hypoactivation during the rest in depression, the observed local decrease of alpha activity after rTMS treatment during the REM sleep confirms the possibility of identifying that EEG frequency as a marker of the cortical activity up-regulation induced by high frequency rTMS as well as a neurophysiological correlate of clinical outcome, providing also an important information regarding the underlying neuronal circuit responsible of antidepressant response. The observed correlation between cortical activity modulation and clinical response indicate clearly that these results are not respectively related to a TMS-induced brain activation independent of the treatment response or to the antidepressant response to rTMS but are determined by their causal and direct relationship. This is the first study, to our knowledge, that combining clinical rTMS-induced effects and neurophysiological evaluation of the EEG sleep changes after a long-term treatment, allows to delineate not only the behavioral outcome, but also the involved sleep cortical circuits, showing that the antidepressant effects of rTMS might be mediated by a significant sleep influence. We conclude that rTMS, and specifically the high frequency stimulation, can represent a relevant strategy in the modulation of hypoactivity syndrome, like in MDD and that the study of the microstructural sleep pattern represents a potential tool for understanding the neurophysiological mechanisms of the mood disorders and their possible regulation. Finally, we propose the left frontal alpha frequency of the REM sleep as a state-dependent marker for depression and its remission.

### Conflict of interest statement

The authors declare that the research was conducted in the absence of any commercial or financial relationships that could be construed as a potential conflict of interest.
